# Identification of a plastidial phenylalanine exporter that influences flux distribution through the phenylalanine biosynthetic network

**DOI:** 10.1038/ncomms9142

**Published:** 2015-09-10

**Authors:** Joshua R. Widhalm, Michael Gutensohn, Heejin Yoo, Funmilayo Adebesin, Yichun Qian, Longyun Guo, Rohit Jaini, Joseph H. Lynch, Rachel M. McCoy, Jacob T. Shreve, Jyothi Thimmapuram, David Rhodes, John A. Morgan, Natalia Dudareva

**Affiliations:** 1 Department of Biochemistry, Purdue University, 175 South University Street, West Lafayette, Indiana 47907-2063, USA; 2 Department of Horticulture and Landscape Architecture, Purdue University, 625 Agriculture Mall Dr, West Lafayette, Indiana 47907-2010, USA; 3 School of Chemical Engineering, Purdue University, 480 Stadium Mall Dr, West Lafayette, Indiana 47907-2100, USA; 4 Bioinformatics Core, Purdue University, 155 South Grant Street, West Lafayette, Indiana 47907, USA

## Abstract

In addition to proteins, L-phenylalanine is a versatile precursor for thousands of plant metabolites. Production of phenylalanine-derived compounds is a complex multi-compartmental process using phenylalanine synthesized predominantly in plastids as precursor. The transporter(s) exporting phenylalanine from plastids, however, remains unknown. Here, a gene encoding a *Petunia hybrida* plastidial cationic amino-acid transporter (PhpCAT) functioning in plastidial phenylalanine export is identified based on homology to an *Escherichia coli* phenylalanine transporter and co-expression with phenylalanine metabolic genes. Radiolabel transport assays show that PhpCAT exports all three aromatic amino acids. *PhpCAT* downregulation and overexpression result in decreased and increased levels, respectively, of phenylalanine-derived volatiles, as well as phenylalanine, tyrosine and their biosynthetic intermediates. Metabolic flux analysis reveals that flux through the plastidial phenylalanine biosynthetic pathway is reduced in *PhpCAT* RNAi lines, suggesting that the rate of phenylalanine export from plastids contributes to regulating flux through the aromatic amino-acid network.

## Abstract (web summary)

Phenylalanine is synthesized in plant chloroplasts and is then exported to the cytosol, where it is a precursor for various secondary metabolites. Here, the authors identify PhpCAT as a plastid phenylalanine transporter required to maintain metabolic flux in petunia.

## Graphical abstract

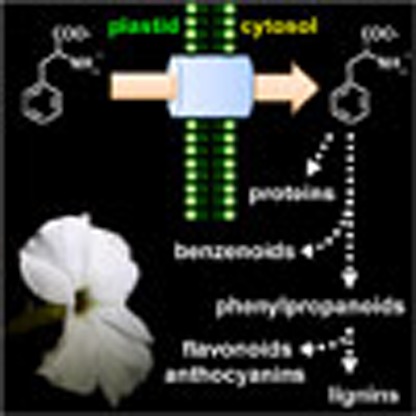

The aromatic amino acid L-phenylalanine is a vital constituent of proteins in all living organisms and serves as a versatile precursor for thousands of indispensable metabolites^1^. In humans, phenylalanine is an essential amino acid obtained through the diet primarily from plant-based sources, either directly or indirectly. Humans can convert phenylalanine to L-tyrosine for incorporation into proteins, as well as to synthesize thyroid hormones^2^ and brain chemicals such as L-dopamine, epinephrine and norepinephrine^3^. In plants, phenylalanine metabolism is even more prevalent and diverse. In fact, plants direct 20–30% of photosynthetically fixed carbon to the production of phenylalanine^4^ and phenylalanine-derived compounds, which constitute approximately 30–45% of plant organic matter^5^ and have profound impacts on growth and development (for example, lignin^6^), reproduction (for example, phenylpropanoids and benzenoids^7^) and defence (for example, salicylic acid^8^, tannins and flavonoids^9^).

Production of phenylalanine-derived compounds in plants is a complex multi-compartmental process relying on phenylalanine mainly synthesized in plastid stroma^10^,^11^,^12^ to first be exported to the cytosol across both the inner and outer plastid envelope membranes. Due to its low permeability coefficient (2.5 × 10^−10^ cm s^−1^ for phenylalanine versus 2 × 10^−4^ cm s^−1^ for water in egg phosphatidylcholine)^13^,^14^, simple diffusion of phenylalanine through membranes cannot meet the cytosolic demand, and therefore its passage is predicted to be protein mediated. In general, the plastid outer envelope is believed to contain pores and channels that confer partial discriminatory transport of metabolites, while the inner membrane, in conjunction with over 100 different transporters, serves as a selective permeability barrier^15^. Within the outer plastid envelope, an outer envelope protein, OEP16 (ref. 16), a member of the plant preprotein and amino-acid transporter superfamily^17^, forms cation-selective high-conductance channels with remarkable permeability for amines and amino acids, including phenylalanine^18^. However, there are still no reports on the characterization of inner envelope amino-acid transporters^19^, despite the presence of several amino-acid biosynthetic pathways in plastids^20^. Phenylalanine is utilized throughout the cell in plant primary and specialized metabolism; thus, its transport across membranes is not limited to plastids. While intercellular amino-acid transporters have been uncovered through recent studies on long-distance nitrogen transport in plants, to date very little is known about intracellular transport of amino acids^21^,^22^,^23^,^24^.

Phenylalanine transport (uptake) has been well studied in microbes. In yeast (*Saccharomyces cerevisiae*) phenylalanine uptake occurs via the broad substrate transporters AGP1 and GAP1, the high-affinity tryptophan permease TAT2, and the branched-chain amino-acid permeases BAP2 and BAP3 (ref. 25). In *Escherichia coli*, phenylalanine uptake proceeds via four distinct systems: (i) PheP (T.C. 2.A.3.1.1), a specific transport system that prefers phenylalanine over tyrosine and does not accept L-tryptophan^26^; (ii) AroP, a general aromatic amino-acid transport system actively transporting phenylalanine, tyrosine and tryptophan with high affinity^27^; (iii) TyrP, a tyrosine-specific system transporting phenylalanine at mM, but not μM, concentrations^28^; and (iv) LIV-I/LS, a branched-chain amino-acid transporter system with broad substrate specificity capable of transporting phenylalanine only at very high concentrations^29^.

The flowers of *Petunia hybrida* cv Mitchell have emerged as an ideal model system for studying plant phenylalanine metabolism. The primary fate of phenylalanine in mature petunia flowers is towards production of cytosolically synthesized volatiles^30^,^31^. However, it has been shown that phenylalanine is predominantly synthesized in plastids^10^,^11^,^12^, implicating the involvement of an unknown transporter to move phenylalanine to the cytosol. Based on homology with *E. coli* PheP and coexpression with phenylalanine metabolic genes, we have identified a gene encoding a *P. hybrida* plastidial cationic amino-acid transporter (PhpCAT) that participates in plastidial phenylalanine transport. Green fluorescent protein (GFP) fusion experiments and subcellular fractionation combined with immunoblotting confirmed that PhpCAT is localized to plastids. Expression of *PhpCAT* was found to be spatially, developmentally and temporally consistent with genes involved in biosynthesis of phenylalanine and phenylalanine-derived volatiles. Radiolabel uptake assays performed using *E. coli-*overexpressing PhpCAT suggest that this transporter is capable of transporting all three aromatic amino acids. RNAi downregulation of *PhpCAT* resulted in reduced emission of phenylalanine-derived volatiles and lowered levels of phenylalanine and tyrosine, and to a lesser extent tryptophan. In contrast, *PhpCAT* overexpression led to increased flux to phenylalanine-derived volatiles and elevated aromatic amino-acid pools. Metabolic flux analysis further showed that when *PhpCAT* is downregulated flux is increased through the alternative phenylpyruvate route, which synthesizes phenylalanine in the cytosol. Based on these data we conclude that PhpCAT is involved in export of phenylalanine from plastids and contributes to controlling carbon flux through the phenylalanine biosynthetic network.

## Results

### *PhpCAT* is a homologue of *E. coli pheP* localized in plastids

With recent advances in gene expression technology, coexpression analysis is becoming an effective tool for discovery of missing genes, including those encoding for plastidial transporters^32^,^33^. Therefore, we took advantage of the fact that petunia flowers synthesize high levels of phenylalanine and phenylalanine-derived volatiles, production of which is coordinately regulated over development and a daily light/dark cycle^10^,^11^,^34^,^35^,^36^,^37^. We generated an RNA-Seq data set based on RNA isolated from corolla tissue harvested at 20:00 h at two developmental stages, day −1 (bud) and day 2 postanthesis (the tissues containing the lowest and highest levels of phenylalanine, respectively^10^) to accurately detect and quantify low-abundance transcripts not represented in our petunia petal-specific EST database^30^ or the Sol genomics network ( http://solgenomics.net). To identify candidate phenylalanine transporters, a tblastn search (cutoff e^−08^) of the petunia RNA-Seq *de novo* assembly transcriptome was performed for genes encoding proteins homologous to *E. coli* PheP^26^. Based on the cutoff, three distinct genes, corresponding to contigs *Ph21511*, *Ph18042* and *Ph19221*, were retrieved and appeared to represent full-length mRNAs encoding proteins comprised of at least 467 amino acids that share 24/41%, 22/43% and 22/41% identity/similarity to *E. coli* PheP, respectively. The cognate proteins of contigs *Ph21511* and *Ph18042* were found to contain 14 and 13 transmembrane helices, respectively, and were annotated by BLAST2GO in the RNA-Seq data sets as putative cationic amino-acid transporters (CATs; T.C. 2.A.3.3.4), which are proton-energized members^38^ of the amino acid–polyamine–organocation transporter family^15^. Contig *Ph19221* was predicted to encode for a putative amino-acid transporter with nine transmembrane domains.

**Figure 1:**
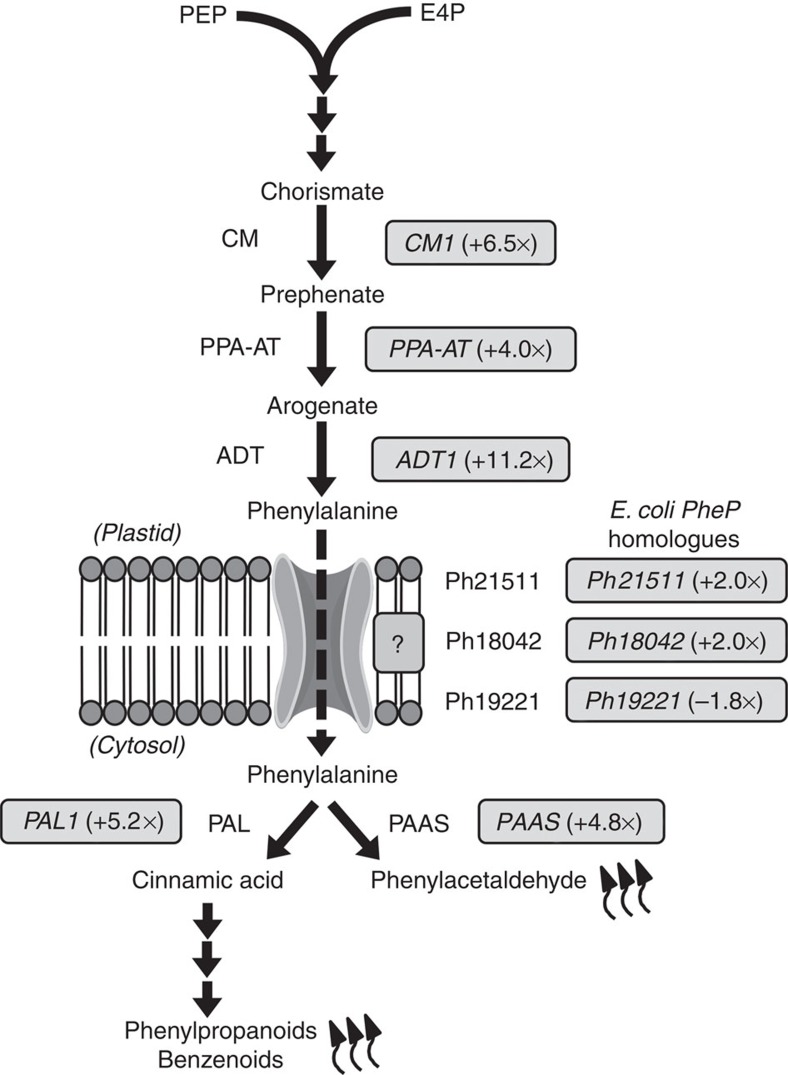
Identification of plastidial phenylalanine transporter candidates. Grey boxes depict average fold change in gene expression in petunia flowers on day 2 postanthesis relative to the bud stage. Changes in expression are shown for petunia homologues of *E. coli pheP*, genes encoding plastidial phenylalanine biosynthetic enzymes chorismate mutase 1 (CM1), prephenate aminotransferase (PPA-AT) and arogenate dehydratase 1 (ADT1), as well as genes for the cytosolic phenylalanine-utilizing enzymes phenylalanine ammonia lyase 1 (PAL1) and phenylacetaldehyde synthase (PAAS). Measurement of gene expression is based on the average number of sequenced fragments±s.e.m. (*n*=3 biological replicates) found for a given contig (gene) per kilobase of sequence per million reads (FPKM) from RNA-Seq analysis of petunia flowers collected at 20:00 h at the bud stage (day −1) and on day 2 postanthesis.

In petunia, it has been shown that expression of phenylalanine metabolic genes increases concomitantly with increased phenylalanine levels^10^,^11^ and emitted volatiles^34^,^35^ as the flower opens. We hypothesized that a phenylalanine transporter may also be transcriptionally upregulated at this time. Since RNA-Seq is a quantitative approach, gene expression on day 2 after flower opening relative to buds was investigated based on the number of counts corresponding to each contig in the generated data sets. The counts are based on the number of reads that map to a given contig normalized for the total number of reads and length of the contig (fragments per kilobase of transcript per million mapped (FPKM) values), which were used by Cufflinks for differential gene expression. First, higher expression of genes encoding plastidial phenylalanine biosynthetic enzymes (chorismate mutase 1, CM1; prephenate aminotransferase, PPA-AT; arogenate dehydratase 1, ADT1) and cytosolic phenylalanine-utilizing enzymes involved in scent formation (phenylalanine ammonia-lyase 1, PAL1; phenylacetaldehyde synthase, PAAS) on day 2 relative to buds (Fig. 1 and Supplementary Table 1) was confirmed to validate the RNA-Seq data sets. Next, expression of the three petunia *pheP* homologues was examined. This analysis revealed that contig *Ph19221* showed an average 1.8-fold decrease in counts on day 2 relative to buds, while contigs *Ph21511* and *Ph18042* both showed a 2-fold average increase (Fig. 1 and Supplementary Table 1). Therefore, only *Ph21511* and *Ph18042* were further examined as plastidial phenylalanine transporter candidate genes.

**Figure 2:**
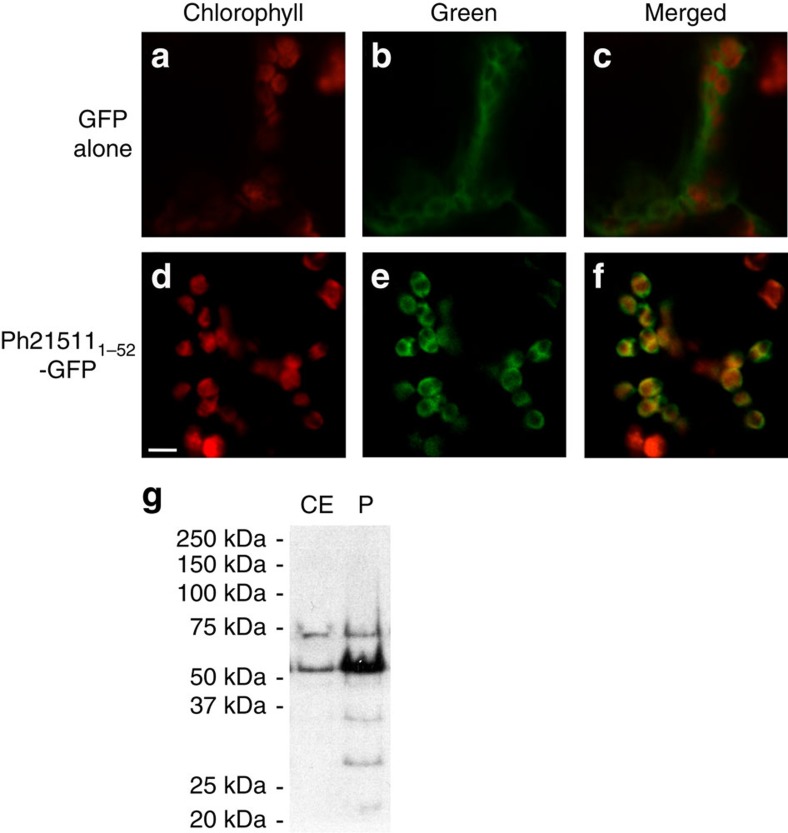
Ph21511 is localized to plastids. (**a–c**) Transient expression of GFP alone in leaves of *Nicotiana benthamiana*. (**d–f**) Transient expression of the first 52 amino acids of Ph21511 fused to the N-terminal end of GFP in leaves of *Nicotiana benthamiana*. Red pseudocolour of chlorophyll autofluorescence is shown in panels **a** and **d**. Green pseudocolour of GFP fluorescence is shown in panels **b** and **e**. Merged images of red and green pseudocolour from **a** and **b** and from **d** and **e** are shown in **c** and **f**, respectively. Scale bar, 5 μm. (**g**) Immunoblot using anti-Ph21511 antibodies against 60 μg protein from total crude extract (CE) and plastids (P) prepared from day 2 petunia flowers.

Recent analysis of the 5,800 transmembrane proteins in *Arabidopsis thaliana* revealed that 660 contained putative amino-terminal plastid transit peptides^15^. Therefore, it was hypothesized that if contigs *Ph21511* and *Ph18042* encode plastidial transporters their cognate proteins should bear detectable N-terminal transit peptides. Analysis of the encoded *Ph18042* protein by multiple subcellular prediction programs (WoLF PSORT: http://www.genscript.com/psort/wolf_psort.html; Predotar: https://urgi.versailles.inra.fr/predotar/predotar.html; TargetP: http://www.cbs.dtu.dk/services/TargetP/) revealed that it does not contain a transit peptide. In contrast, the first 43 amino acids of the encoded *Ph21511* protein were predicted with a score of 0.837 by TargetP to serve as a plastid-targeting signal. To experimentally test for plastidial localization, the first 52 amino acids of Ph21511, which include all the residues prior to the first predicted transmembrane domain, were fused to the N-terminus of the GFP reporter protein and transiently expressed in tobacco leaves (Fig. 2a–f). Whereas the GFP control displayed fluorescence confined to the cytosol (Fig. 2a–c), the green fluorescence of the Ph21511_1-52_-GFP construct resulted in co-localization with the red autofluorescence of chlorophyll in plastids (Fig. 2d–f). To independently verify the confocal microscopy result, total crude extracts and isolated plastids were prepared from 2-day-old petunia flowers and examined by immunoblotting using purified anti-Ph21511 antibodies. The apparent molecular mass of the detected protein, which migrated above the 50-kDa marker, from both crude extract and the plastid fraction was consistent with the calculated size (57.9 kDa) for mature (that is, without transit peptide) Ph21511 (Fig. 2g). Moreover, the detected signal from equal amounts (60 μg) of loaded protein was drastically enriched in the plastid fraction compared to crude extract (Fig. 2g), thus confirming that Ph21511 is targeted to plastids. Based on the experimentally determined localization and the work presented below, the protein encoded by *Ph21511* was designated as PhpCAT for *P. hybrida* plastidial cationic amino-acid transporter.

### *PhpCAT* is developmentally and rhythmically regulated

**Figure 3:**
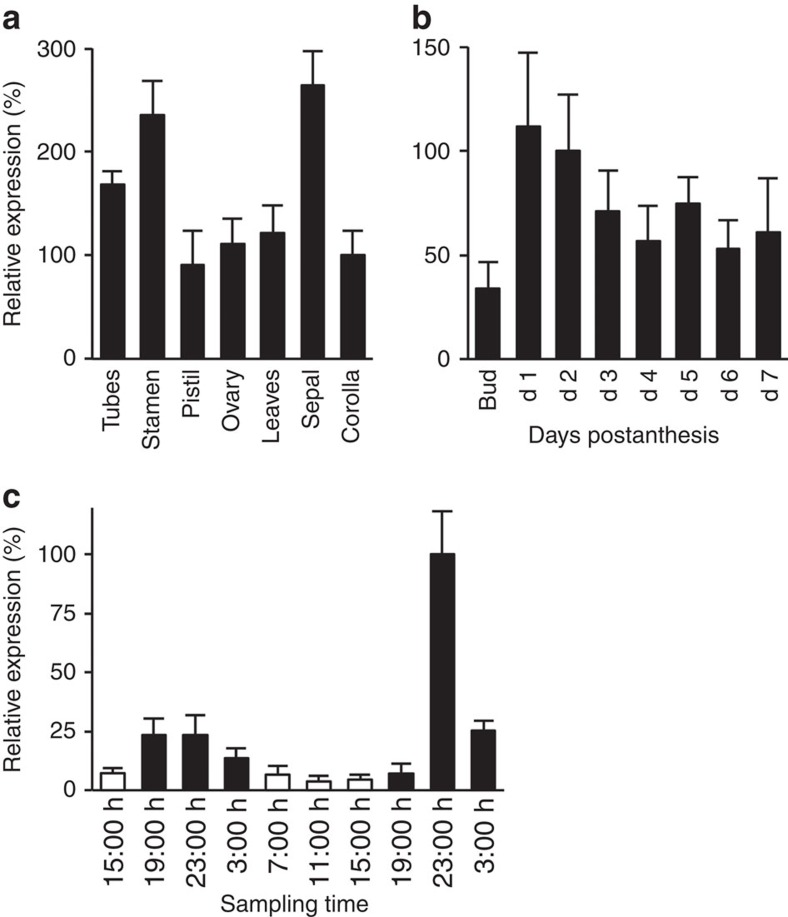
Expression profiles of *PhpCAT*. (**a**) Tissue-specific expression of *PhpCAT* in leaves and floral organs at 15:00 h on day 2 postanthesis relative to corolla set at 100%. (**b**) Developmental *PhpCAT* expression profile at 15:00 h in petunia flowers starting from the bud stage and on days 1 through 7 postanthesis relative to expression on day 2 set at 100%. (**c**) Rhythmic changes in *PhpCAT* expression in corollas of petunia flowers from day 1 to day 3 postanthesis during a normal light/dark cycle shown relative to expression at 23:00 h on day 2 set at 100%. All transcript levels were determined by qRT-PCR and obtained relative to the reference gene (*elongation factor 1-α*). Data are means ± s.e.m. (*n*=3 biological replicates).

Petunia flowers predominantly emit high levels of phenylalanine-derived volatiles at night^30^,^39^. To accomplish this, petunia flowers have evolved synchronized developmental and temporal expression patterns for genes involved in synthesis of phenylalanine^10^,^11^,^36^ and phenylalanine-derived volatiles^34^,^35^,^37^. Investigation of *PhpCAT* transcript levels by quantitative reverse transcription (qRT)-PCR with gene-specific primers revealed that it is expressed in all flower organs, as well as in leaves (Fig. 3a). Moreover, *PhpCAT* expression was lowest at the bud stage (day −1) and increased upon flower opening (Fig. 3b), displaying a typical pattern for petunia flower genes involved in biosynthesis of phenylalanine^10^,^11^,^36^ and phenylalanine-derived scent compounds^34^,^35^,^37^ (Fig. 1 and Supplementary Table 1). Remarkably, *PhpCAT* was also found to exhibit rhythmic expression over a daily light cycle, with the highest transcript abundance occurring at night on day 2 postanthesis (Fig. 3c), which corresponds to the time of highest emission of phenylalanine-derived volatiles^30^,^39^. Thus, the developmental and temporal expression of *PhpCAT* is consistent with that of a transporter *a priori* needed to export phenylalanine out of plastids for production of floral volatiles.

### PhpCAT transports all three aromatic amino acids

**Figure 4:**
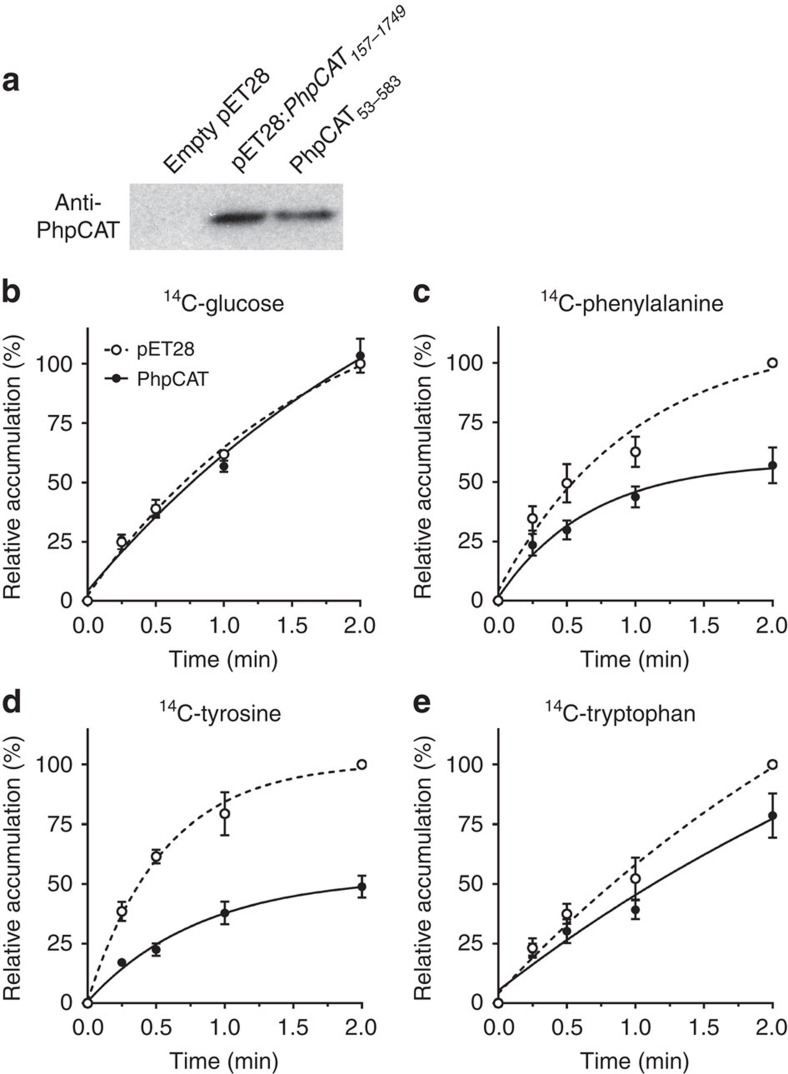
PhpCAT aromatic amino-acid transport activity. (**a**) Immunoblots on 2.5 μg of whole-cell crude extracts prepared from *E. coli* carrying an empty pET28a vector or codon-optimized pET28a:*PhpCAT*_*157-1749*_, and 2.5 μg of purified PhpCAT_53-583_. (**b–e**) Transport assays on whole *E. coli* cells carrying empty pET28a (white circles) or pET28a:*PhpCAT*_*157-1749*_ (black circles). An equal number of cells were incubated with 100 μM ^14^C-glucose as a negative control (**b**), ^14^C-phenylalanine (**c**), ^14^C-tyrosine (**d**) or ^14^C-tryptophan (**e**) for the indicated time periods until termination of the assays by rapid filtration. Data are presented as a percentage relative to the corresponding empty-vector control value at 2 min set to 100%. Data are means±s.e.m. (*n*≥3 biological replicates). Two-tailed Student's *t-*tests revealed that relative accumulation of radiolabelled aromatic amino acids was statistically different in *E. coli*-expressing *PhpCAT* versus empty-vector control (*P ≤* 0.01).

The use of whole-cell uptake assays in a heterologous *E. coli* expression system has been an informative approach for elucidating the biochemical function of plastid transporters^40^,^41^,^42^,^43^,^44^,^45^. To determine if PhpCAT is capable of transporting phenylalanine and/or the other aromatic amino acids, uptake assays with ^14^C-phenylalanine, ^14^C-tyrosine and ^14^C-tryptophan were performed with intact *E. coli* cells expressing recombinant PhpCAT_53-583_ (N-terminal 6XHis-tagged PhpCAT without its predicted transit peptide) or carrying an empty vector as a control. An immunoblot using purified anti-PhpCAT (anti-Ph21511) antibodies against *E. coli* crude extracts verified that recombinant PhpCAT_53-583_ was expressed in isopropyl-1-thio-β-D-galactopyranoside (IPTG)-induced cells carrying the pET28a:*PhpCAT*_*157-1749*_ construct, but not empty pET28a vector (Fig. 4a). As a negative control, ^14^C-glucose was used to test if overexpression of PhpCAT affected endogenous *E. coli* uptake activities. First, glucose was selected because it was not expected to be a substrate of PhpCAT. In addition, one of the native glucose transport systems is proton driven similar to the *E. coli* AroP uptake system, which is responsible for 80–90% of aromatic amino-acid transport. Since no difference in ^14^C-glucose uptake was detected between *E. coli* harbouring the empty vector and cells expressing PhpCAT_53-583_, this indicates that proton-driven metabolite transport, including the endogenous aromatic amino-acid uptake system, across the membrane was not perturbed (Fig. 4b). On the other hand, *E. coli* cells expressing PhpCAT_53-583_ accumulated significantly lower levels of ^14^C-phenylalanine (Fig. 4c), ^14^C-tyrosine (Fig. 4d) and, to a lesser extent, ^14^C-tryptophan (Fig. 4e), compared to the empty-vector control. The detected levels of radiolabelled amino acids result from the net difference between uptake into and efflux out of the cell. Since no decrease in uptake via the endogenous aromatic amino acid *E. coli* transport systems is presumably occurring, the reduced accumulation of aromatic amino acids in cells expressing PhpCAT_53-583_ is due to efflux via PhpCAT. These results demonstrate that all three aromatic amino acids are substrates of PhpCAT.

### PhpCAT links plastidial–cytosolic phenylalanine metabolism

**Figure 5:**
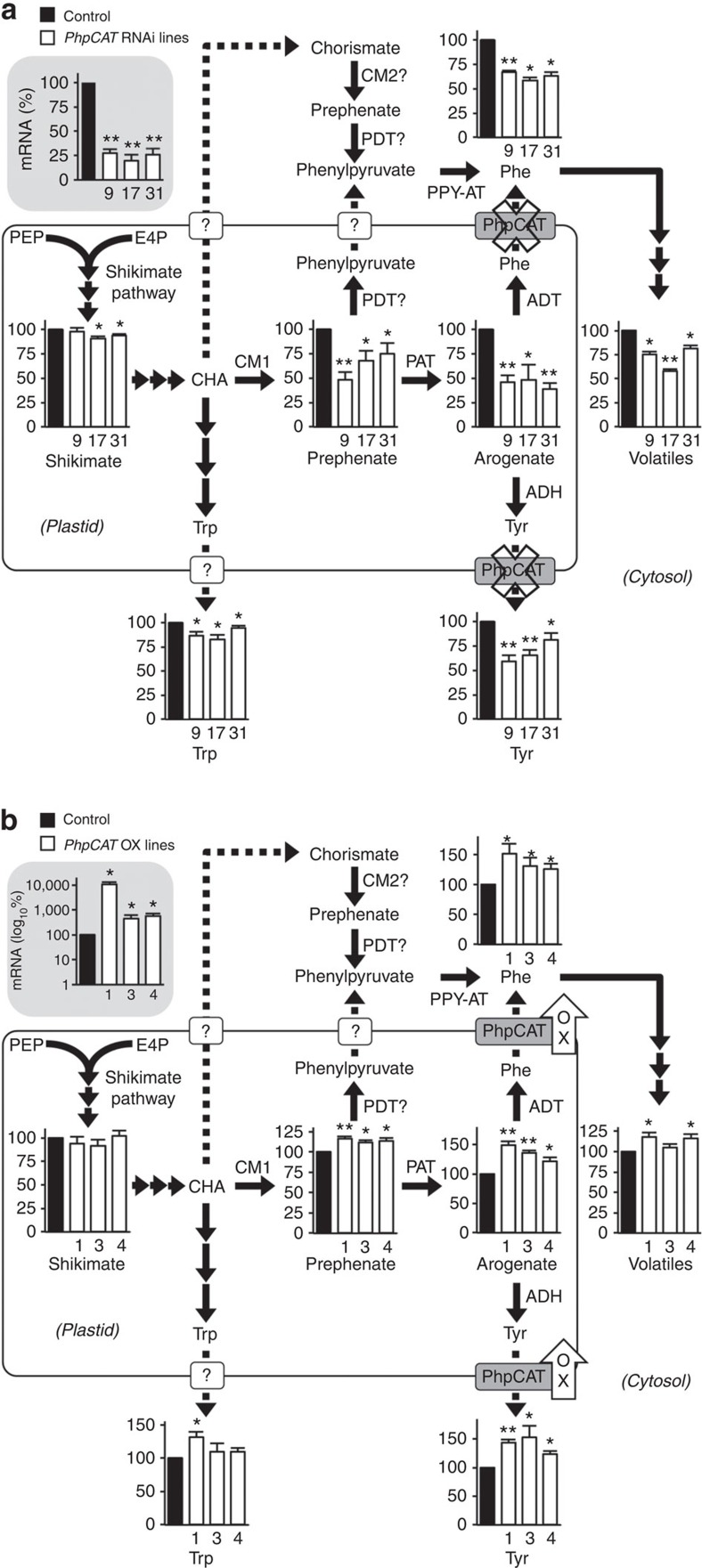
Metabolic profiling of petunia flowers from *PhpCAT*-RNAi and *PhpCAT*-overexpression lines. (**a**) Effects of *PhpCAT* RNAi suppression. (**b**) Effects of *PhpCAT* overexpression. *PhpCAT* mRNA levels (*n*=3 biological replicates), internal pools of shikimate, prephenate, arogenate, phenylalanine, tyrosine and tryptophan (*n*=4), and total emission of phenylalanine-derived volatiles (*n*≥3) were measured in three independent *PhpCAT*-RNAi downregulated and *PhpCAT*-overexpression lines (white bars) and compared to control values (black bars), which were set at 100%. Data are means±s.e.m. *P<0.05, **P<0.01 by two-tailed Student's *t*-tests. Dotted lines indicate trafficking steps. White boxes with question marks indicate unknown transporters/transport steps. In (**a**) the ‘x' over PhpCAT depicts downregulation. In (**b**) the arrow with ‘OX' depicts overexpression. ADH, arogenate dehydrogenase; ADT, arogenate dehydratase; CHA, chorismate; CM, chorismate mutase; E4P, erythrose 4-phosphate; PPA-AT, prephenate aminotransferase; PDT, prephenate dehydratase; PEP, phosphenolpyruvate; PPY-AT, phenylpyruvate aminotransferase.

To examine if PhpCAT functions *in vivo* as an exporter of phenylalanine from plastids, *PhpCAT* expression was downregulated using an RNAi strategy under control of a petal-specific promoter^46^. Three independent lines with 75–80% downregulated *PhpCAT* transcript levels (Fig. 5a) were selected for subsequent detailed metabolic profiling. Moreover, immunoblot analysis of crude extracts and purified plastid fractions from RNAi line 17 with purified anti-PhpCAT (anti-Ph21511) antibodies indicated that PhpCAT was downregulated at the protein level (Supplementary Fig. 1). Consistent with an *in planta* role in plastidial phenylalanine export, downregulation of *PhpCAT* led to 20–42% reduction in total emission of phenylalanine-derived volatiles relative to control (Fig. 5a), but the extent of decrease varied for each compound (Supplementary Fig. 2). Phenylalanine and tyrosine levels were also decreased in *PhpCAT*-RNAi lines by up to 42% compared to control (Fig. 5a), which was likely due to reduced amounts of their biosynthetic intermediates prephenate (25–52% decreased in *PhpCAT*-RNAi lines versus control) and arogenate (54–61% decreased in *PhpCAT*-RNAi lines versus control) (Fig. 5a). At the same time, only minimal decreases were observed in the pool sizes of tryptophan (5–18% reduced) and shikimate (up to 9% reduced) in *PhpCAT*-RNAi lines compared to control (Fig. 5a).

To further investigate the role of PhpCAT *in vivo*, *PhpCAT* was overexpressed under the control of a petal-specific promoter^44^. Three different lines showing 4.5–100-fold increase in *PhpCAT* transcript levels (Fig. 5b) were chosen for further metabolite analysis. Total emission of phenylalanine-derived volatiles in detached flowers increased by up to 18% in *PhpCAT* overexpressors compared to control (Fig. 5b), although the degree of increase differed for individual compounds (Supplementary Fig. 3). In addition, the levels of phenylalanine and tyrosine increased from 23 to 53% in *PhpCAT* overexpressors compared to control (Fig. 5b), likely as a result of elevated pools of phenylalanine biosynthetic intermediates prephenate and arogenate (11–16% and 22–48% increase in *PhpCAT* overexpressors versus control, respectively) (Fig. 5b). Moreover, the tryptophan pool displayed an increasing trend and no changes were detected in shikimate levels in *PhpCAT* overexpressors compared to control (Fig. 5b). Taken together, these data indicate that PhpCAT is involved in plastidial phenylalanine export *in planta*. Moreover, profiling of plastidial phenylalanine biosynthetic intermediates suggests that PhpCAT is involved in regulating flux through the aromatic amino-acid biosynthetic network.

### PhpCAT controls phenylalanine biosynthetic flux

**Figure 6:**
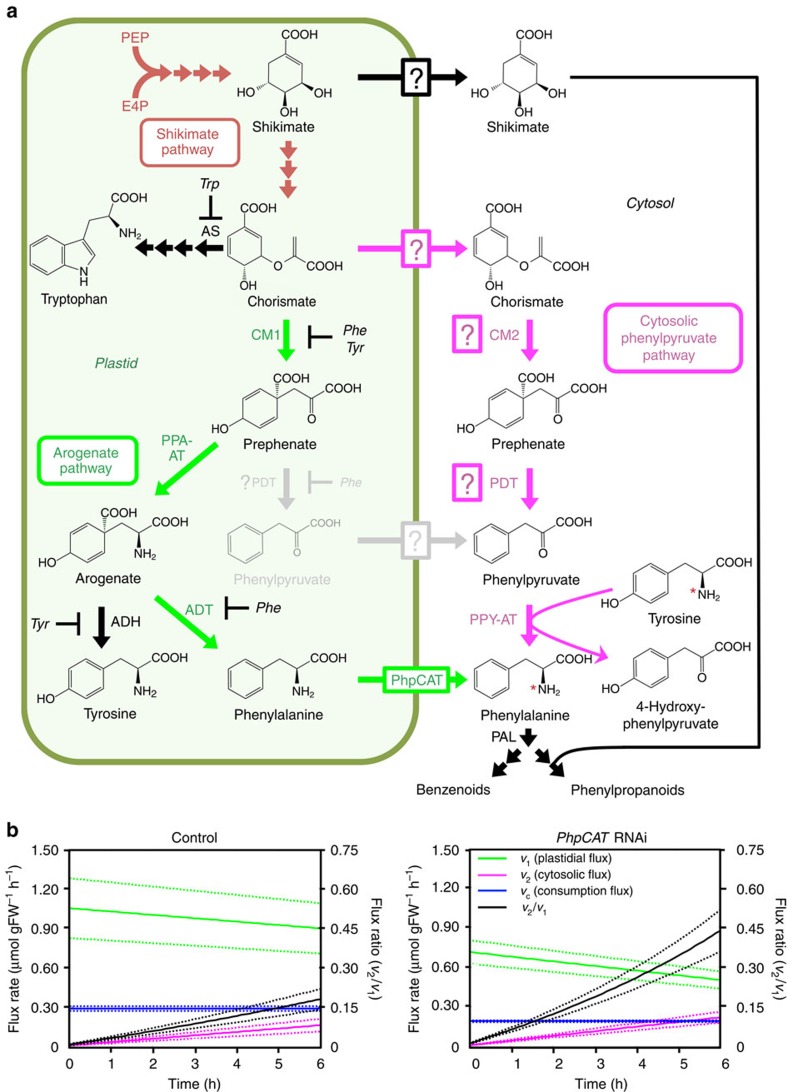
Metabolic modelling of the phenylalanine biosynthetic network in control and *PhpCAT*-RNAi lines. (**a**) Scheme depicting cytosolic formation of phenylalanine and potential feedback inhibition mechanisms involved in plastidial phenylalanine biosynthesis^1^. In *PhpCAT*-RNAi lines, phenylalanine and tyrosine may accumulate in plastids and feedback inhibit arogenate dehydratase (ADT) and arogenate dehydrogenase (ADH), respectively, as well as chorismate mutase 1 (CM1). If plastidial ADTs also function as prephenate dehydratases (PDTs), then the enzymatic conversion of prephenate to phenylpyruvate may also be subject to feedback inhibition by phenylalanine. It is unclear if phenylpyruvate utilized in the alternative phenylalanine biosynthetic pathway originates from the plastid, or from cytosolic conversion of chorismate exported from the plastid. White boxes with question marks indicate unknown transporters/transport steps. AS, anthranilate synthase. For remaining abbreviations, see Fig. 5. (**b**) Flux models representing the phenylalanine biosynthetic network in 2-day-old petunia flowers from control and *PhpCAT-*RNAi lines. Computer-assisted metabolic modelling was performed using pool sizes and isotopic abundances of phenylalanine and tyrosine, and measurements of phenylalanine-derived volatile emission from petunia petal tissue supplied with 10 mM ^15^N-tyrosine for up to 6 h. The rate of plastidial flux (*v*_1_) in *PhpCAT*-RNAi lines was found to be significantly lower than control based on comparisons of absolute fluxes and two-tailed Student's *t-*tests corrected by Bonferroni method (*P*<0.05). The rate of change in cytosolic flux (*v*_2_) in *PhpCAT*-RNAi lines was found to be significantly higher than control (*P*<0.01) based on paired-sample two-tailed Student's *t-*tests of slopes (see Methods for additional details). *n*=3 for control and *n*=6 for PhpCAT-RNAi lines.

Since downregulation of *PhpCAT* led to a decrease in the levels of phenylalanine, tyrosine, and their shared precursors prephenate and arogenate (Fig. 5a), we hypothesized that phenylalanine and tyrosine accumulate inside plastids of *PhpCAT*-RNAi lines and feedback inhibit the arogenate pathway (Fig. 6a). Recently we showed that plants also contain an alternative pathway that proceeds via phenylpyruvate to produce phenylalanine in the cytosol, and flux through this route increases when the plastidial biosynthetic pathway (via arogenate) is impaired^12^. In the alternative pathway, a cytosolic phenylpyruvate aminotransferase preferentially converts phenylpyruvate to phenylalanine using tyrosine as an amino donor^12^ (Fig. 6a). Interestingly, tyrosine cannot serve as an amino donor for PPA-AT in the plastidal arogenate pathway^11^. Taking advantage of this characteristic to distinguish between the two pathways, we employed metabolic flux analysis with stable isotopic labelling using ^15^N-tyrosine to determine the effect of reduced plastidial phenylalanine export on carbon flux through the parallel phenylalanine biosynthetic pathways.

Excised 2-day-old petunia flowers from control and *PhpCAT*-RNAi lines 9 and 17 were fed with 10 mM ^15^N-tyrosine starting at 18:00 h, harvested after 2, 4 and 6 h, and analysed by liquid chromatography-mass spectrometry (LC-MS) to determine phenylalanine and tyrosine pool sizes and isotope abundances. Similar to what was observed previously^12^, the labelling percentage and concentration of phenylalanine increased linearly over 6 h, as did the level of emitted volatiles (Supplementary Fig. 4). To assess the control PhpCAT exerts on metabolic fluxes through the phenylalanine biosynthetic network, a metabolic flux model was developed (see Methods). The simulation revealed that *v*_*1*_, the rate of synthesis through the plastidial arogenate pathway, was 32 and 44% lower (*P*<0.05, Student's *t*-test, *n*≥3) at *t*_0_ and *t*_6_, respectively, in *PhpCAT*-RNAi lines compared to control (Fig. 6b and Supplementary Table 2). This finding is consistent with our hypothesis that reduced export of phenylalanine and tyrosine from plastids in *PhpCAT*-RNAi lines leads to feedback inhibition of the arogenate pathway. At the same time, the flux analysis showed that in control and the *PhpCAT-*RNAi lines, *v*_2_, the flux through the cytosolic phenylpyruvate pathway, was minor at *t*_0_, but significantly increased over the 6-h period (Fig. 6b and Supplementary Table 2). In addition, increase in the rate of *v*_2_ in *PhpCAT* knockdowns was more rapid than in control (*P*<0.01, Student's *t*-test, *n*≥3) (Fig. 6b and Supplementary Table 2), suggesting that more carbon flux is directed through the cytosolic pathway. Taking this into account with the decrease in flux through the arogenate pathway, the relative contribution of cytosolic phenylalanine production is considerably higher in *PhpCAT-*RNAi lines compared to control (a *v*_2_/*v*_1_ flux ratio of 0.44 versus 0.18, respectively, at *t*_6_) (Fig. 6b and Supplementary Table 2).

## Discussion

Eukaryotic metabolic networks are often spread throughout multiple subcellular compartments separated by organellar membrane barriers, through which precursors, intermediates and end products must pass. While some metabolites may passively diffuse across membranes, others require protein-mediated transport, as is the case for phenylalanine synthesized in plastids. The current study took advantage of the distinctive transcriptional developmental regulation of phenylalanine metabolism in petunia flowers to identify a plastidial amino-acid transporter, PhpCAT, which is capable of transporting all three aromatic amino acids (Fig. 4). Increases and decreases in the levels of phenylalanine-derived volatiles, as well as in phenylalanine, tyrosine, and their biosynthetic intermediates prephenate and arogenate in *PhpCAT* overexpression and RNAi lines, respectively (Fig. 5), support an *in vivo* role for PhpCAT as a plastidial aromatic amino-acid exporter. Moreover, flux through the phenylpyruvate pathway, which produces phenylalanine in the cytosol, was increased when *PhpCAT* was downregulated (Fig. 6b).

It is likely that while the total cellular pools of phenylalanine and tyrosine decrease in *PhpCAT*-RNAi lines compared to control (Fig. 5a), these amino acids accumulate inside the plastid and feedback inhibit the shikimate and/or aromatic amino-acid biosynthetic pathways. Disproportional effects on the concentrations of phenylalanine, tyrosine, and their precursors prephenate and arogenate compared to shikimate and tryptophan (Fig. 5a) may point to feedback inhibition of CM1 (Fig. 6a) in *PhpCAT*-RNAi lines. Previously it was shown that petunia CM1 activity is not inhibited at the same concentration (50 μM) of phenylalanine and tyrosine^36^ known to regulate *Arabidopsis* CM1 (ref. 47). However, in light of the high demand for phenylalanine in petunia flowers, and the fact that inhibition constants (*K*_i_) of other plant CMs are in the range of 0.3–1.1 mM^1^, it is possible that petunia CM1 is inhibited at higher concentrations of phenylalanine. To test this, we assayed preparations of petunia plastids for CM activity in the presence of a range of phenylalanine concentrations (Supplementary Figs 5 and 6). Indeed, petunia plastidial CM activity was found to be inhibited by phenylalanine with an IC_50_ of 748 μM (Supplementary Fig. 6). Thus, the metabolic phenotype on the arogenate pathway observed in *PhpCAT-*RNAi lines (Fig. 5a) could be attributed to feedback inhibition of petunia CM1. Conversely, in *PhpCAT-* overexpression lines, levels of phenylalanine, tyrosine, prephenate and arogenate were increased compared to control (Fig. 5b), which may indicate reduced levels of phenylalanine and tyrosine in plastids leading to the relaxation of naturally occurring feedback regulation of their biosynthetic enzymes, including CM1. The fact that tryptophan also trended to increase in *PhpCAT* overexpression lines compared to control (Fig. 5b) might further imply reduced feedback restrictions on tryptophan biosynthesis and/or the shikimate pathway.

Downregulation of *PhpCAT* expression in petunia flowers by 75–80% led to 20–42% reduction in the emission of phenylalanine-derived volatiles (Fig. 5a). The smaller reduction in volatiles relative to *PhpCAT* expression is likely the result of one or a combination of several factors. (i) It is possible that the remaining amount of PhpCAT transporter activity was sufficient to sustain the observed volatile emission rate. (ii) In addition or alternatively, there could be other specific and/or non-specific amino-acid transporters, which are not homologous to *E. coli* PheP, involved in exporting phenylalanine from plastids. (iii) Another possibility is the contribution of a phenylalanine storage pool in the vacuole to the production of phenylalanine-derived products. Vacuoles, which are separated from cytosol by a single, semi-permeable membrane called the tonoplast, are involved in temporary and long-term storage of numerous metabolites^48^, including free phenylalanine^49^. In recent years, tonoplast-localized transporters have been discovered in plants and shown to be an integral part of a complex cellular network controlling plant metabolism^48^. Two *Arabidopsis* amino-acid transporters, AtCAT2 and AtCAT4, have been shown to localize to the vacuolar membrane^22^,^23^. Though the physiological function of AtCAT4 is still unknown, AtCAT2 has been shown to regulate free amino-acid levels in *Arabidopsis* leaves^22^. Interestingly, AtCAT2 is the most similar *Arabidopsis* CAT homologue to Ph18042 (Supplementary Fig. 7), the other *E. coli* PheP homologue with increased gene expression in petunia flowers on day 2 postanthesis (Fig. 1 and Supplementary Table 1). Similar to AtCAT2, Ph18042 is also predicted to localize to the vacuole and it seems conceivable that it may contribute to modulating cytosolic phenylalanine levels in petunia flowers. (iv) Finally, in *PhpCAT*-RNAi lines the phenylpyruvate pathway could partially compensate for the shortage of phenylalanine being exported from the plastid. Recently it was shown that flux through the cytosolic phenylpyruvate pathway (Fig. 6a) increases when entry into the arogenate pathway is genetically blocked^12^. Similarly, the present study shows that the phenylpyruvate pathway has higher relative contribution when plastidial phenylalanine export is impeded in *PhpCAT*-RNAi lines (Fig. 6b). It still remains an open question at which upstream step flux is redirected towards the phenylpyruvate pathway. It is possible that CM1 is feedback inhibited by phenylalanine and tyrosine accumulated in plastids of *PhpCAT*-RNAi lines; thus, flux may be redirected at the level of chorismate (Fig. 6a). Plants contain a cytosolic isoform of CM, CM2, with higher affinity for chorismate than their plastidial CM1 counterparts^1^, which may produce prephenate in the cytosol for the alternative phenylalanine biosynthetic pathway (Fig. 6a). This scenario implicates the involvement of a plastidial chorismate transporter and cytosolic prephenate dehydratase (PDT), neither of which has been discovered. Alternatively, phenylpyruvate may be generated in the plastid via dehydration/decarboxylation of prephenate by moonlighting ADT (or an unidentified PDT) and then exported through an unknown transporter to the cytosol (Fig. 6a). However, *PhpCAT*-RNAi lines have reduced prephenate levels and the catalytic efficiency of PPA-AT^11^ is much higher than those of the known ADTs with moonlighting PDT activity^10^. Thus, together these factors likely preclude formation of a sufficient quantity of phenylpyruvate in plastids to sustain the cytosolic pathway.

The occurrence of a plastidial CAT transporter does not appear to be unique to petunia. Phylogenetic reconstruction with the *Arabidopsis* CAT family reveals that the closest homologues of PhpCAT are CAT7 (73% identity/85% similarity) and AtCAT6 (73% identity/84% similarity) (Supplementary Fig. 7). While *Arabidopsis* CAT7 has unknown function, it possesses a putative transit peptide predicted to serve as a plastid-targeting signal^23^. On the other hand, AtCAT6 has previously been shown to function in sink tissues as a transporter of amino acids, including phenylalanine^38^. Fusion of GFP to the N-terminus of AtCAT6 revealed that it is localized to the plasma membrane^38^. However, it should be further investigated if AtCAT6 is dual localized, because similar to *Arabidopsis* CAT7 and PhpCAT, it contains a predicted transit peptide^23^ (with a TargetP score of 0.949).

At the cellular level, transporters are integral parts of metabolic networks as they mediate interactions between multiple pathways across different subcellular compartments. Metabolic flux analysis performed in the current study strongly supports the idea that transporters, such as PhpCAT, further exert control over fluxes through metabolic networks by influencing organellar metabolite concentrations, which in turn biochemically regulate enzymes. Thus, development of organellar metabolite-profiling techniques^50^,^51^, coupled with stable isotope labelling, will be needed to further dissect the role PhpCAT plays in feedback control of the aromatic amino-acid network.

## Methods

### Chemicals andreagents

D-[^14^C(U)] glucose (300 mCi mmol^−1^), L-[^14^C(U)] phenylalanine (350 mCi mmol^−1^), L-[^14^C(U)] tyrosine (482 mCi mmol^−1^) and L-[side chain-3-^14^C] tryptophan (55 mCi mmol^−1^) were from American Radiolabeled Chemicals (St Louis, MO). All other chemicals were from Sigma-Aldrich ( http://www.sigmaaldrich.com) unless otherwise noted.

### Plant material and growth conditions

Wild-type and transgenic *P. hybrida* cv. Mitchell diploid plants (W-115; Ball Seed Co., West Chicago, IL) were grown under standard greenhouse conditions^44^ with a light period from 18:00 until 21:00 h. For the *PhpCAT*-RNAi construct, DNA containing two spliced *PhpCAT* cDNA fragments of the coding region corresponding to nucleotides 30-577 and 30-368, the latter in antisense orientation to create a hairpin structure, was synthesized (Genscript, Piscataway, NJ). 5′-EcoRI and 3′-BamHI sites were added for directional subcloning into pRNA69 containing the *Clarkia breweri linalool synthase* (*LIS*) petal-specific promoter^44^. The resulting cassette containing the *LIS* promoter and the synthetic *PhpCAT* hairpin fragment was cut out using SacI/NotI and subcloned into the pART27 simpler binary vector^52^. For the *PhpCAT* overexpression construct, *PhpCAT* was amplified using the forward primer 5′-GAATTCATGGAGACCCATAGCTCCTCTTTCTCTAACATAAA-3′ and reverse primer 5′-GGATCCTTACACTTTGAGAGTATGGTCCTGGTTTTCAATAG-3′ and directionally subcloned into the EcoRI and BamHI sites (underlined in the respective primers) of pRNA69 in frame with the *LIS* promoter. The resulting cassette was cut out with SacI/NotI and subcloned into the pART27 binary vector. The final *PhpCAT*-RNAi and *PhpCAT* overexpression constructs were used for *Agrobacterium tumefaciens* (strain GV3101)-mediated transformation of *P. hybrida* cv. Mitchell diploid using the standard leaf disk transformation method^53^.

### Generation of RNA-Seq data sets

RNA was isolated from corollas of at least eight wild-type *P. hydrida* cv. Mitchell flowers (per biological replicate) at 20:00 h on day −1 (bud) and day 2 postanthesis using an RNeasy plant mini kit (Qiagen, Valencia, CA). RNA was treated with DNase I to eliminate genomic DNA using the TURBODNA-free kit (Ambion, Foster City, CA). A total of six cDNA libraries, representing three biological replicates of each developmental stage, were constructed using an mRNA-Seq Kit (Illumina, San Diego, CA), and 100-bp paired-end reads were generated using Illumina HiSeq2500 at the Purdue Genomics Center, with at least 35 million reads per library. Sequences were first assessed for quality by FastQC (v. 0.10.0; http://www.bioinformatics.babraham.ac.uk). Trimming was done using FASTX toolkit (v. 0.0.13; http://hannonlab.cshl.edu/fastx_toolkit/), parameters were configured to trim reads less than Phred33 score of 30 and resulting reads had to be at least 50 bases long to be retained. After quality trimming, *de novo* assembly of the transcriptome was performed using Trinity^54^ (v. R 2012-10-05) with default parameters. Trinity generated 100,419 unique contigs (≥500 bp), which were then used as reference transcriptome for RNA-seq analysis and were annotated using BLAST2GO^55^ (version 2.5.0) using default parameters. The N50 for these contigs was 2,509 bp, which was calculated by identifying that contig length in which 50% of the assembly's total bases are included in contigs of equal or greater length, and 50% of the assembly's total bases are included in contigs of lesser length. A total of 25,603 contigs were ≥N50 (Supplementary Fig. 8). The quality-trimmed reads were mapped against the generated transcriptome using Tophat (v. 2.0.6; http://tophat.cbcb.umd.edu/) with default parameters. The total number of reads from all samples that unambiguously mapped to a gene feature was 217,939,059 (93.34%). Each assembled contig in the *de novo* transcriptome assembly was treated as a putative gene, and therefore gene counts for differential expression analysis were aggregated based on contig counts, using HTSeq (v.0.5.3p7; http://www-huber.embl.de/users/anders/HTSeq/). Counts for each sample were merged using custom perl scripts and used for downstream differential gene expression analysis. Although we acknowledge FPKM values may be affected, we did not attempt to collapse or reduce contigs prior to read mapping and differential gene expression analysis in order to avoid potential false negatives.

Differential expression analysis between samples ‘bud' and ‘day2' was performed using three different methods, of which two were using ‘R' (v. 2.15.2; http://www.r-project.org/). Basic exploration of the read count data file was performed to ensure data quality. An edgeR object was created by combining the counts matrix, library sizes and experimental design using edgeR (v. 3.0.3) package^56^. Normalization factors were calculated for the counts matrix, followed by estimation of common dispersion of counts. Exact test for differences between the negative binomial distribution of counts for the two experimental conditions resulted in finding differential expression, which was then adjusted for multiple hypothesis testing. Another method named ‘voom' from limma package^57^ (v. 3.14.1) was also used for differential expression analysis. The function of ‘voom' carries out log_2_ transformation of counts followed by mean-variance estimation and assigning weight to each transformed value. Linear model coefficients were then calculated using limma's design matrix, contrast matrix and log_2_ transformed values. Linear model was fitted using empirical Bayes method and differences between counts for two experimental conditions were calculated, which were then adjusted for multiple hypothesis testing. Additionally, mapping of each sample to the transcriptome using TopHat resulted in .bam files, which were then used by the Cufflinks (v 2.0.2) suite of programs^58^. In all, 96.98% of the contigs found to be differentially expressed by Cufflinks were also differentially expressed by both edgeR and voom.

### qRT-PCR

Sample collection, RNA isolation and qRT-PCR were performed as previously described^35^. Briefly, samples were collected from the tissues indicated in the text and RNA was isolated using the RNeasy Plant Mini Kit (Qiagen). Total RNA was treated with DNaseI to eliminate genomic DNA using the TURBO DNA-free Kit (Ambion) and was reverse-transcribed to cDNA using the High Capacity cDNA Reverse Transcription Kit (Applied Biosystems). *PhpCAT* was amplified using the specific primers 5′-AGCACTTTCCGATACCCCAA-3′ and 5′-GAACTCGGCTTGTTTCTTCGA-3′. *PhpCAT* expression was analysed relative to the reference gene *elongation factor 1-α* which was amplified using primers 5′-CCTGGTCAAATTGGAAACGG-3′and 5′-CAGATCGCCTGTCAATCTTGG-3′. Each data point represents an average of three independent biological samples.

### Subcellular localization of PhpCAT

The first 156 nucleotides encoding the 52 amino acids preceding the first predicted transmembrane domain of *PhpCAT* (*Ph21511*) were amplified using the forward primer 5′-CACCATGGAGACCCATAGCTCCTCTTTCTCTAACATAAA-3′ and reverse primer 5′-CCGTTTCATATCACCACCAGACCG-3′. The resulting fragment was subcloned into pENTR^TM^/D-TOPO (Invitrogen^TM^, Carlsbad, CA), sequence verified and transferred into pK7FWG2^59^ via Gateway^TM^ technology, resulting in an in-frame fusion with the N-terminal end of GFP. The Ph21511_1-52_-GFP construct and an empty GFP vector were transformed into *A. tumefaciens* strain GV3101, infiltrated into *Nicotiana benthamiana* leaves, and imaged 48 h later. Images were acquired using a Zeiss LSM710 laser spectral scanning confocal microscope with a C-Apochromat 40 × /1.20 W objective (Zeiss, Thornwood, NY). GFP was excited with an argon laser at wavelength 488 nm and emissions were collected over a 493–598-nm bandpass. Chlorophyll fluorescence was excited by a HeNe laser at wavelength 633 nm and emissions were collected over a 647–721-nm bandpass.

### Heterologous expression of PhpCAT and transport assays

The coding sequence of *PhpCAT* was synthesized (Genscript, Piscataway, NJ) with codon optimization for expression in *E. coli* using GenScript's OptimumGene™ Gene Design system. A truncated version (minus the predicted transit peptide) of codon-optimized *PhpCAT* was amplified with the forward primer 5′-CATATGAGTCTGCGTTGGTACGACCTGGTT-3′ and reverse primer 5′-CTCGAGTTAGACTTTCAGGGTATGGTCTTGGTTTTCA-3′, which introduced NdeI and XhoI sites (underlined), respectively, for directional cloning into pET28a (Novagen) in frame with an N-terminal 6XHis-tag. The resulting codon-optimized pET28a:*PhpCAT*_*157-1749*_ construct was sequence verified and transformed into Lemo21(DE3) *E. coli* (New England Biosciences, Ipswich, MA). Starter cultures containing kanamycin and chloramphenicol were used to inoculate 25 ml of Luria-Bertani (LB) at an *A*_600_ of 0.05. When *A*_600_ reached approximately 0.4, IPTG was added to a final concentration of 0.5 mM and incubation was continued for 2 h at 18 °C. Cells were harvested by centrifugation, and nickel-affinity purification of PhpCAT_53-583_ under denaturing conditions was performed according to *The QIAexpressionist*^*TM*^ (5th edn, 2003) protocols 10 and 17.

For the radiolabel uptake assays, IPTG induction and preparation of cells carrying empty pET28a or codon-optimized pET28a:*PhpCAT*_*157-1749*_ was performed as described above except that cells were washed and resuspended to an *A*_600_ of 4.0 in M9 minimal media. To initiate the assays, 100 μl of prepared cells was added to 100 μl of M9 incubation media containing ^14^C-labelled glucose (0.25 μCi), phenylalanine (0.35 μCi), tyrosine (0.35 μCi) or tryptophan (0.2 μCi). Concentrations of substrates were pre-adjusted in the M9 incubation media to 200 μM with cold substrate. Uptake assays were carried out at 30 °C in 1.5 ml Eppendorf microfuge tubes and terminated after the indicated time periods by rapid filtration of the reaction through a 0.45-μm Whatman filter (GE Healthcare Life Sciences, Pittsburgh, PA) under vacuum. Filters were washed two times with 1 ml M9 media and then transferred into a 20-ml scintillation vial containing 10 ml of EcoLite(+) scintillation cocktail (MP Biochemicals, Santa Ana, CA). Radioactivity in the samples was quantified by scintillation counting.

### Metabolic flux analysis with ^15^N-tyrosine labelling

We generated a metabolic flux model that utilizes experimentally determined pool sizes and isotopic enrichment of phenylalanine from exogenously fed ^15^N-tyrosine. Feeding of 10 mM ^15^N-tyrosine (Cambridge Isotope Laboratories, Andover, MA) to 2-day-old corollas of control and *PhpCAT*-RNAi lines was performed as previously described^12^. Similar to our previous labelling study^12^, the labelling percentage of tyrosine quickly reached >80% and a pseudo-steady state within 2 h upon feeding, and stayed constant for the rest of the experimental period. The model was based on the dynamic mass balance around phenylalanine as defined as


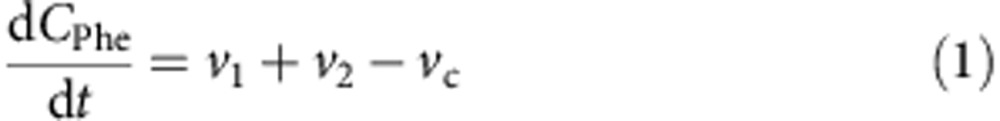


where *C*_Phe_ is the phenylalanine pool size, *v*_1_ is the flux through the plastidial arogenate pathway, *v*_2_ is the flux through the cytosolic phenylpyruvate pathway and *v*_c_ is the consumption rate of phenylalanine. To determine *v*_2_, it was also required to know the mass balance of isotopic enrichment for phenylalanine as defined as


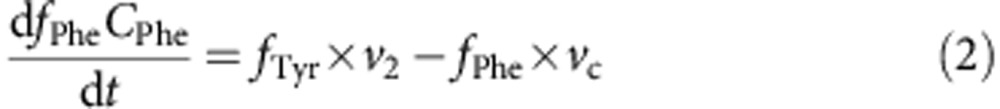


where *f*_Phe_ and *f*_Tyr_ represent isotopic abundance of phenylalanine and tyrosine, respectively, in the total pools. With equations (1) and (2), *v*_1_ and *v*_2_ can be derived as follows:


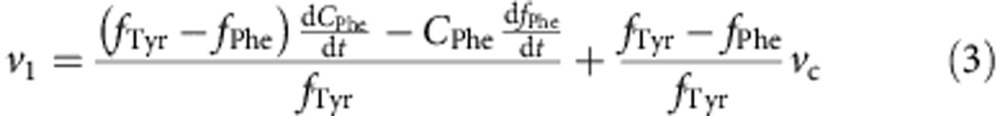


and


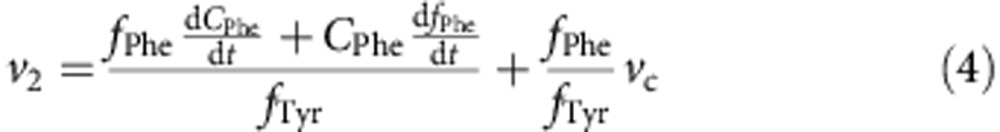


Since the labeling percentage and concentration of phenylalanine increased linearly over 6 h, 
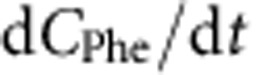
 and 
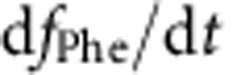
 were computed from the slopes of the time-series data. Then every 6-min estimates of *C*_Phe_ and *f*_Phe_ were derived based on 
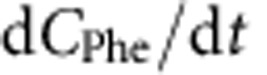
 and 
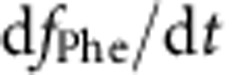
. *f*_Tyr_ was the average labelling percentage of tyrosine along the experiment. To determine the control *v*_c_, emitted volatiles from day 2 control flowers fed with 10 mM tyrosine were collected for 2, 4 and 6 h starting at 18:00 h. No statistical differences were found in the scent profiles of flowers fed with 10 mM tyrosine compared to control. Since the volatile amount was found to increase linearly (Supplementary Fig. 4b), *v*_c_ was assumed to be constant during the experimental period, and was derived from the slope of the time-series data. The *v*_c_ for *PhpCAT* RNAi lines was subsequently determined by multiplying the control *v*_c_ by the average fractional decrease of total emission observed in lines 9 and 17 (Fig. 5a). With these obtained values, *v*_1_ and *v*_2_ were calculated along the experimental period every 6 min using equations (3) and (4). The whole simulation was performed in Matlab R2013a environment (The MathWorks, Inc., Natick, MA). Variances in the estimated slopes were derived with a standard linear regression procedure as described^60^, while setting the intercepts as constants was based on experimental measurements. Since flux values are a function of the estimated trend slopes and other experimental measurements, flux variances can be derived by considering the propagation of errors based on the following equation:


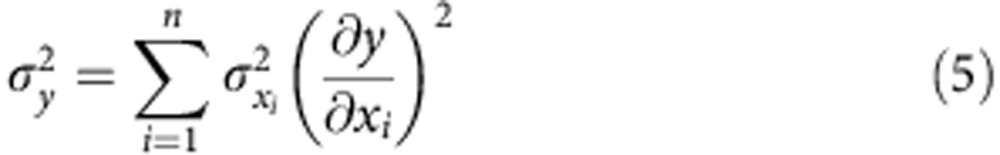


where *y*=*f*(*x*_1_,…,*x*_n_).

### Preparation of plastids

Plastids were isolated from approximately 25 g of 1–3-day-old petals of control, *PhpCAT*-RNAi line 9 and *PhpCAT* overexpression line 3 petunia plants. Flowers were kept in the dark for 2 h prior to plastid preparations to deplete starch. Petals were placed in a chilled blender containing 200 ml ice-cold medium A (0.5 M sorbitol, 10 mM MES/NaOH pH 6.3, 4 mM Na-ascorbate, 4 mM cysteine, 2 mM MgCl_2_, 1.5 mM KH_2_PO_4_, 1 mM MnCl_2_, 1 mM EDTA-Na_2_) and homogenized with three 2-s pulses. The resulting homogenate was filtered through two layers of Miracloth (Calbiochem, La Jolla, CA). An aliquot of filtrate (crude extract) was taken from each preparation and set aside, and the remainder was centrifuged at 6,000 *g*, 4 °C for 5 min. The pellet was washed twice in medium B (0.33 M sorbitol, 10 mM MES/NaOH pH 7.6), resuspended in 2 ml of medium B, and layered over a discontinuous Percoll^TM^ gradient consisting of 80% (2 ml) and 25% (6 ml) Percoll^TM^ prepared in medium B. After centrifugation (9,200 *g*, 4 °C for 20 min in a swinging bucket rotor with no brake applied), the intact plastid fractions (approximately 1.5 ml) were collected from the interface of the Percoll^TM^ layers, washed two times in medium B, and finally resuspended in 50 mM NaH_2_PO_4_ (pH 8.0) with 5% (v/v) glycerol, flash frozen and stored at −80 °C.

### Immunoblots

Immunoblot analysis was performed essentially as described previously^61^ using petunia crude extracts and plastids, *E. coli* crude extracts, and the purified PhpCAT_53-583_. Protein sample concentration was determined by the Bio-Rad Bradford protein assay. To solubilize membrane proteins, protein-loading dye containing 10% SDS was added to protein samples, which were then incubated at room temperature for 3 h, and centrifuged at 5,000*g* for 5 min to pellet debris. Immunodetection was performed on 2.5 μg protein using purified rabbit anti-PhpCAT polyclonal antibodies (1:3,000) generated against a synthetic peptide CMIDPDAPFSGAFMG (Genscript, Piscataway, NJ; http://www.genscript.com/custom-polyclonal-antibody-production-services.html). A goat anti-rabbit IgG horseradish peroxidase conjugate (1:30,000) was used as secondary antibody (Bio-Rad, Hercules, CA; 170-6515). Antigen bands were visualized using an enhanced chemiluminescence reagent (PerkinElmer, Waltham, MA) according to the manufacturer's protocol, and exposing the gels on Eastman Kodak X-OMART AR film. An uncropped immunoblot corresponding to Fig. 4a is shown in Supplementary Fig. 9.

### Chorismate mutase assays

CM assays were performed using protein prepared from petunia plastids according to a previously published method^62^. Briefly, corolla tissue was collected from day 2 flowers at 21:00 h, immediately frozen in liquid nitrogen, ground with mortar and pestle, and lyophilized at −80 °C, 0.01 mbar for a minimum of 72 h. Approximately 0.3 g of dried tissue was resuspended in 2.5 ml heptane/tetrachloroethylene (density=1.32 g ml^−1^) and homogenized in a ball mill. The resulting suspension was layered atop a freshly prepared heptane/tetrachloroethylene density gradient, then centrifuged for 90 min at 13,000*g*, 4 °C. The resolved gradient was divided into six fractions; each fraction was divided into half and all solvent evaporated under nitrogen flow. For enzyme assays, the resulting residue was resuspended in 1 ml of protein resuspension buffer (50 mM Tricine-NaOH, 10% glycerol, pH 8.4). Insoluble debris was pelleted by centrifugation, and the supernatant desalted into protein resuspension buffer before use in assays. Marker assays were completed for plastids (NADP-glyceraldehyde-3-phosphate dehydrogenase) and cytosol (alcohol dehydrogenase) as described previously^63^,^64^. Plastid content peaked in the second heaviest fraction (7–12 ml as measured from the bottom of the gradient, approximate density 1.49–1.45 g ml^−1^), as 46% of total recovered GAPDH activity was found in that fraction, compared to 18% of ADH activity. This fraction was used for subsequent analysis.

CM assays were performed as described previously^10^, except that they were adapted to high-performance liquid chromatography detection as follows. Following reaction incubation and subsequent conversion of prephenate to phenylpyruvate by acid hydrolysis, reactions were neutralized by addition of 50 μl of 1 M NaOH, and 10 μl of the reaction mixture was analysed by high-performance liquid chromatography using the Waters Atlantis dC18 column (3 μm, 2.1 × 150 mm) held at 35 °C, with an 8-min linear gradient of 5–70% acetonitrile in 1% formic acid at a flow rate of 0.4 ml min^−1^. Phenylpyruvate and chorismate were detected by absorbance at 288 nm and quantified by comparison to authentic standards. For phenylpyruvate product confirmation, MS/MS was completed using an Agilent 6460 Triple Quadrupole LC-MS. Data were collected for product ion scans on a precursor *m*/*z* of 163.1 in negative mode using a collision energy of 20 V, a fragmentor voltage of 70 V and dwell time of 50 ms. The jet stream ESI interface had a gas temperature of 325 °C, gas flow rate of 8 l min^−1^, nebulizer pressure of 35 psi, sheath gas temperature of 250 °C, sheath gas flow rate of 7 l min^−1^, capillary voltage of 3,000 V and nozzle voltage of 500 V.

### Targeted metabolite profiling

Petunia volatiles (benzaldehyde, benzyl alcohol, phenylacetaldehyde, methylbenzoate, phenylethanol, benzylacetate, eugenol, isoeugenol, vanillin, benzylbenzoate and phenylethylbenzoate) were collected from detached flowers of control, *PhpCAT*-RNAi and *PhpCAT* overexpression lines (minimum of three flowers per biological replicate) from 18:00 to 22:00 h on day 2 postanthesis by a closed-loop stripping method and analysed by gas chromatography-MS as previously described^44^. Internal pools of the aromatic amino acids and organic acids (shikimate, prephenate and arogenate) from control and transgenic tissues collected (minimum of eight flowers per biological replicate) at 20:00 h on day 2 postanthesis were quantified by gas chromatography- or LC-MS as previously described^10^.

## Additional information

**Accession codes:** The GenBank accession number for the PhpCAT sequence mentioned in this article is KP694324. The petunia RNA-seq data set has been deposited in NCBI Gene Expression Omnibus (GEO) under accession code GSE70948.

**How to cite this article:** Widhalm, J. R. *et al.* Identification of a plastidial phenylalanine exporter that influences flux distribution through the phenylalanine biosynthetic network. *Nat. Commun.* 6:8142 doi: 10.1038/ncomms9142 (2015).

## Supplementary Material

Supplementary Information Supplementary Figures 1-9, Supplementary Tables 1-2 and Supplementary References
