# Supplementary material for: Identification of a plastidial phenylalanine exporter that influences flux distribution through the phenylalanine biosynthetic network
Source: Nat Commun. 2015 Sep 10;6:8142. doi: 10.1038/ncomms9142 (PMC4647861; doi:10.1038/ncomms9142)
Supplement: Supplementary Information — Supplementary Figures 1-9, Supplementary Tables 1-2 and Supplementary References [file ncomms9142-s1.pdf]

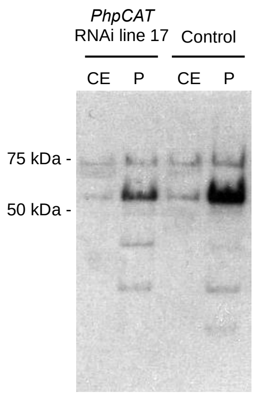

Supplementary Figure 1 | Immunoblot using anti-PhpCAT (anti-Ph21511) antibodies against 60 µg protein isolated from crude extract (CE) and plastids (P) of day 2 petunia flowers from control and *PhpCAT*-RNAi line 17. Mature PhpCAT is predicted to be 57.9 kDa.

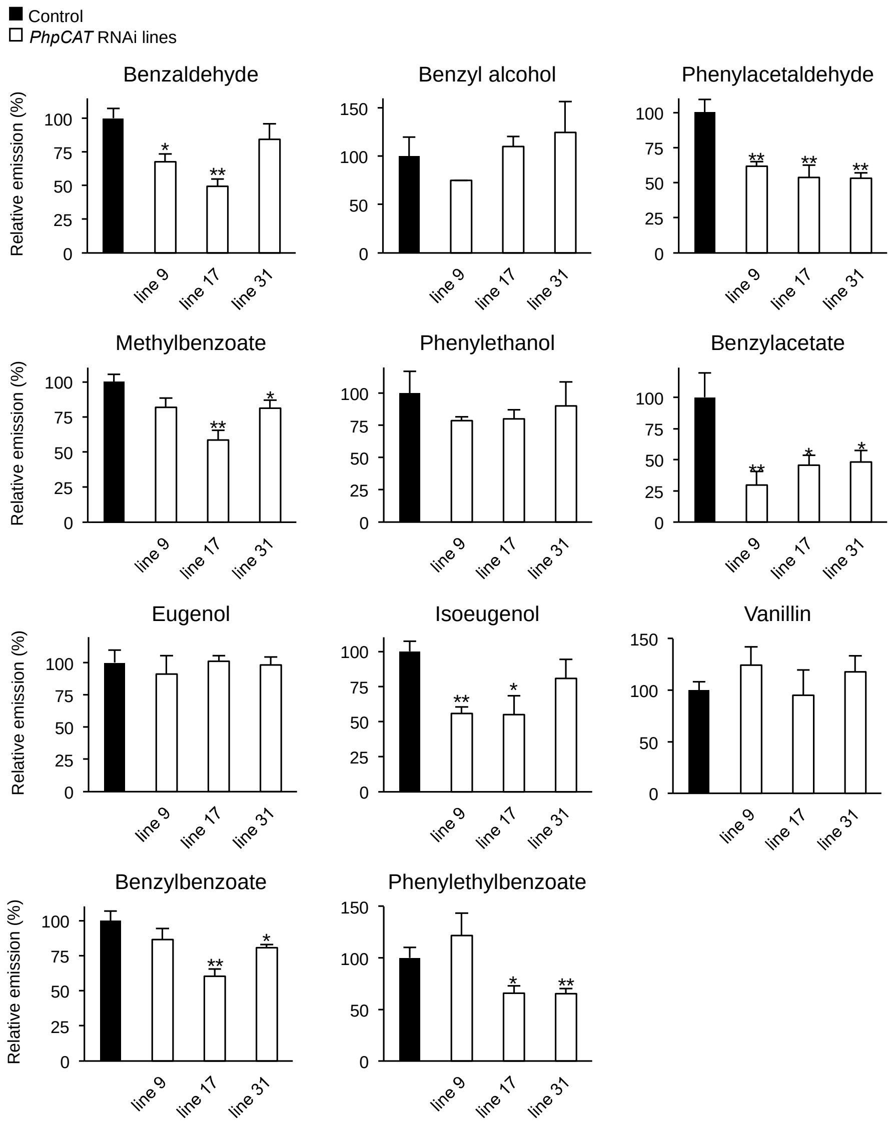

Supplementary Figure 2 | Effect of *PhpCAT* RNAi suppression on emission of individual phenylalanine-derived volatiles from petunia petals.

Volatiles were collected from 6:00-10:00 PM on day 2 postanthesis and are presented as percentages of levels in wild-type set at 100%. Data are means ± s.e.m. ($n \geq 3$ biological replicates). * = $p < 0.05$, ** = $p < 0.01$ by two-tailed Student's *t*-tests.

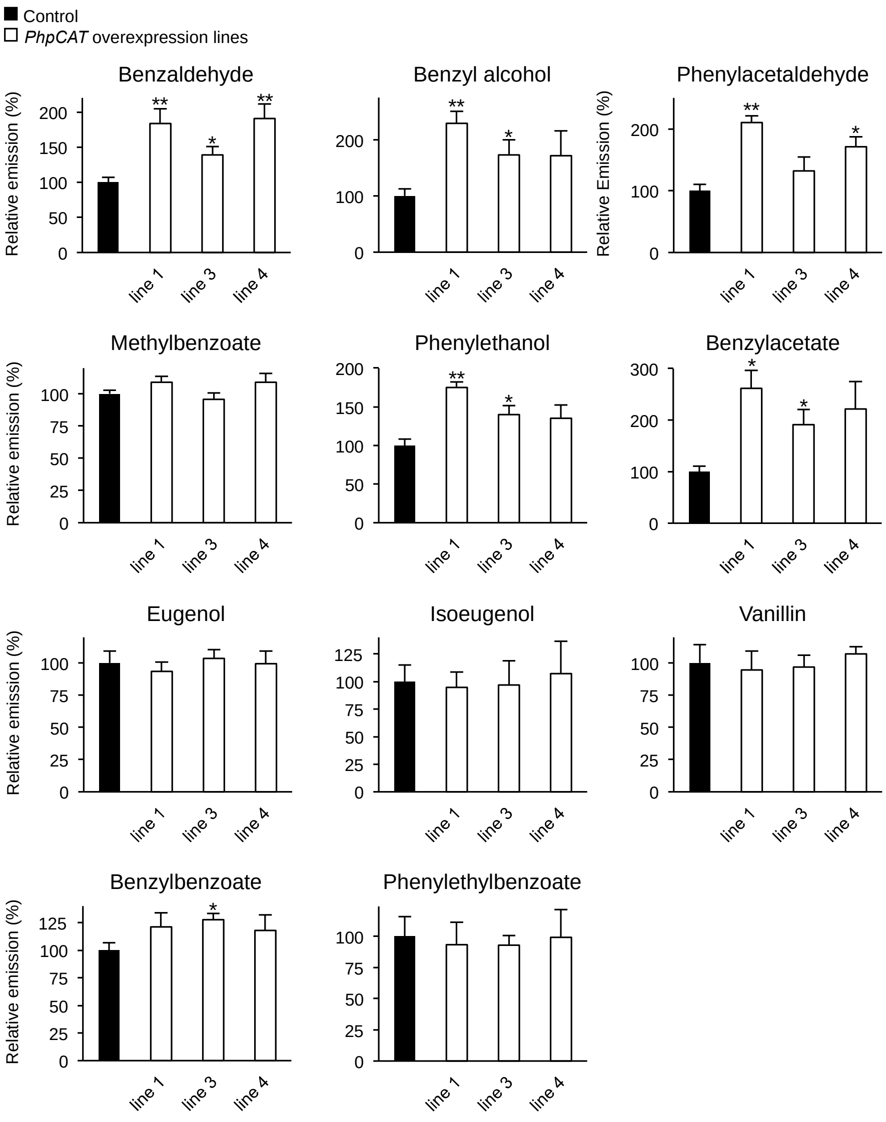

Supplementary Figure 3 | Effect of *PhpCAT* RNAi overexpression on emission of individual phenylalanine-derived volatiles from petunia petals.

Volatiles were collected from 6:00-10:00 PM on day 2 postanthesis and are presented as percentages of levels in wild-type set at 100%. Data are means ± s.e.m. ($n \geq 3$ biological replicates). * = $p < 0.05$, ** = $p < 0.01$ by two-tailed Student's *t*-tests.

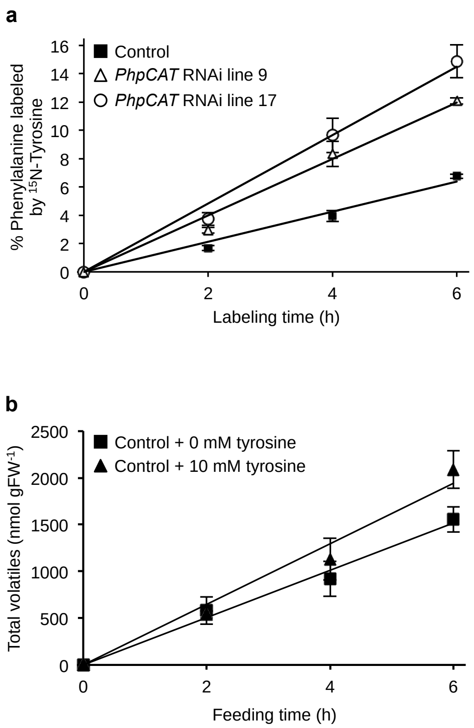

Supplementary Figure 4 | Effect of feeding (^{15}N-)tyrosine on the labeling of phenylalanine and the levels of phenylalanine-derived volatiles.

(**a**) Phenylalanine is more highly labeled by ^{15}N-tyrosine in *PhpCAT*-RNAi lines than control. 10 mM ^{15}N-tyrosine was fed to 2-day-old petunia flowers from control (solid square) and *PhpCAT*-RNAi lines 9 (open triangle) and 17 (open circle) for 2, 4, and 6 h beginning at 6 PM. Labeling of phenylalanine pools were analyzed by TOF LC-MS. Data are means ± s.e.m. (*n* = 3 biological replicates, except for control 6-h time point, *n* = 2). Incorporation of ^{15}N-label into phenylalanine was linear over the 6-h time course (R^2 = 0.9834, 0.9858, and 0.9895 for control and *PhpCAT*-RNAi lines 9 and 17, respectively). (**b**) Feeding with tyrosine does not effect the emission rate of phenylalanine-derived volatiles. Two-day-old wild-type petunia flowers were fed with 0 (black squares) or 10

mM tyrosine (black triangles) for 2, 4, and 6 h beginning at 6 PM. The total amount of phenylalanine-derived volatiles emitted was analyzed by GC-MS. Data are means ± s.e.m. (*n* = 3 biological replicates). The total amount of volatiles released remained constant over the 6-h time course (R^2 = 9878 and 0.9746 for control and 10 mM tyrosine-fed flowers, respectively), with no significant differences in slopes.

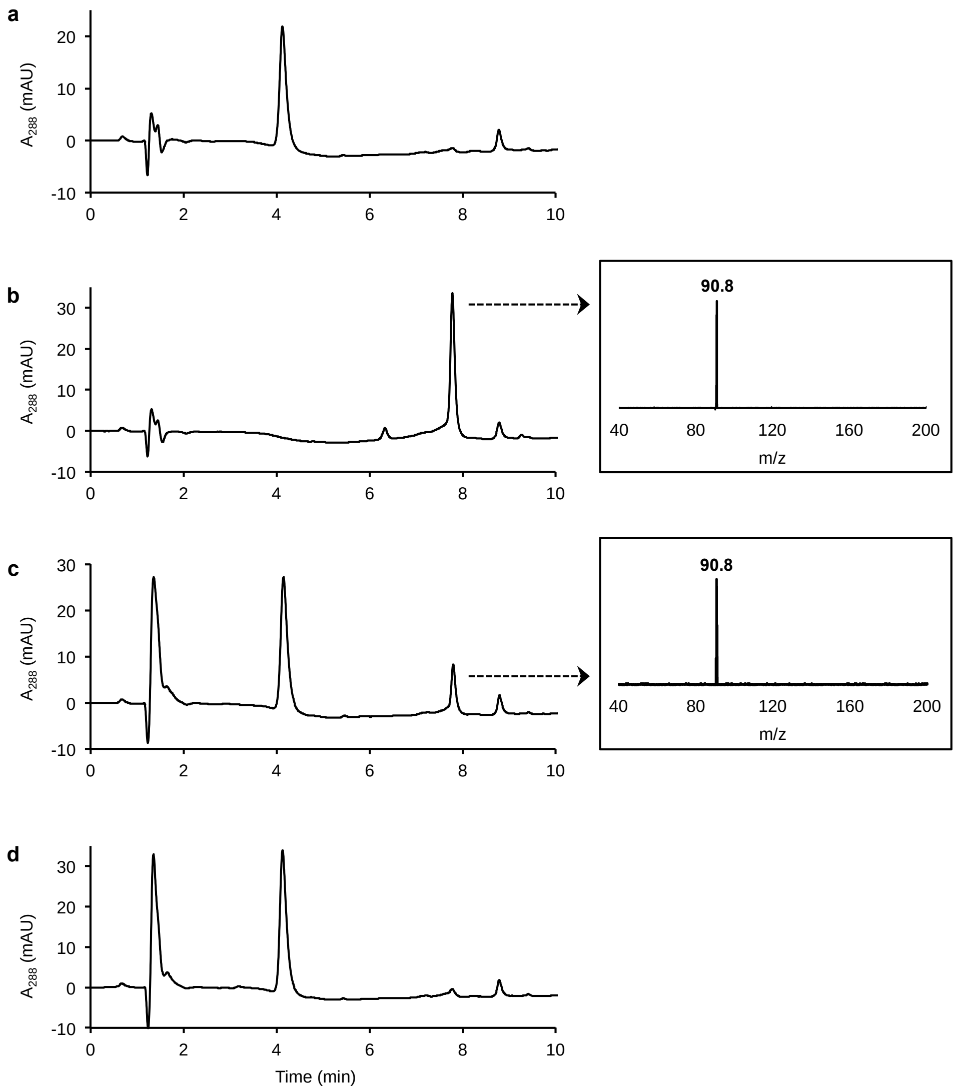

Supplementary Figure 5 | HPLC assays of chorismate mutase activity in desalted extracts from petunia flower plastids.

(a) Authentic chorismate standard (1 nmol). **(b)** Authentic phenylpyruvate standard (1 nmol). **(c)** Assay in the absence of phenylalanine inhibitor. **(d)** Lack of product formed when desalted plastid extract was boiled before addition to assay. Plastids were isolated from day 2 petunia flowers at 9 PM. Box insets show the dominant ion of phenylpyruvate (90.8 m/z) detected by Triple Quadrupole LC-MS/MS analysis. Exact masses displayed are $(M-H)^-$.

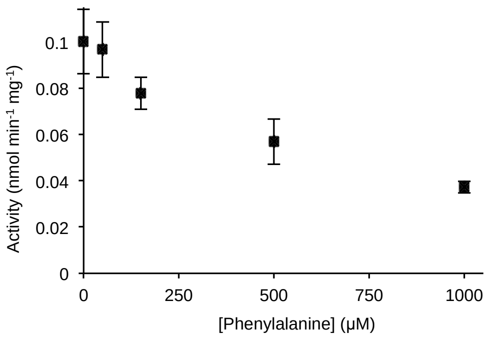

Supplementary Figure 6 | Phenylalanine inhibition of chorismate mutase activity in desalted petunia plastid extracts.

Chorismate mutase activity was measured in desalted plastid extracts with the presence of various phenylalanine concentrations. Activity was measured by acidifying the reaction after incubation to convert enzymatically-formed prephenate to phenylpyruvate, which was quantified by HPLC spectrophotometry. Plastids were isolated from day 2 petunia flowers at 9 PM. Data are means ± s.e.m. (n = 3 replicates).

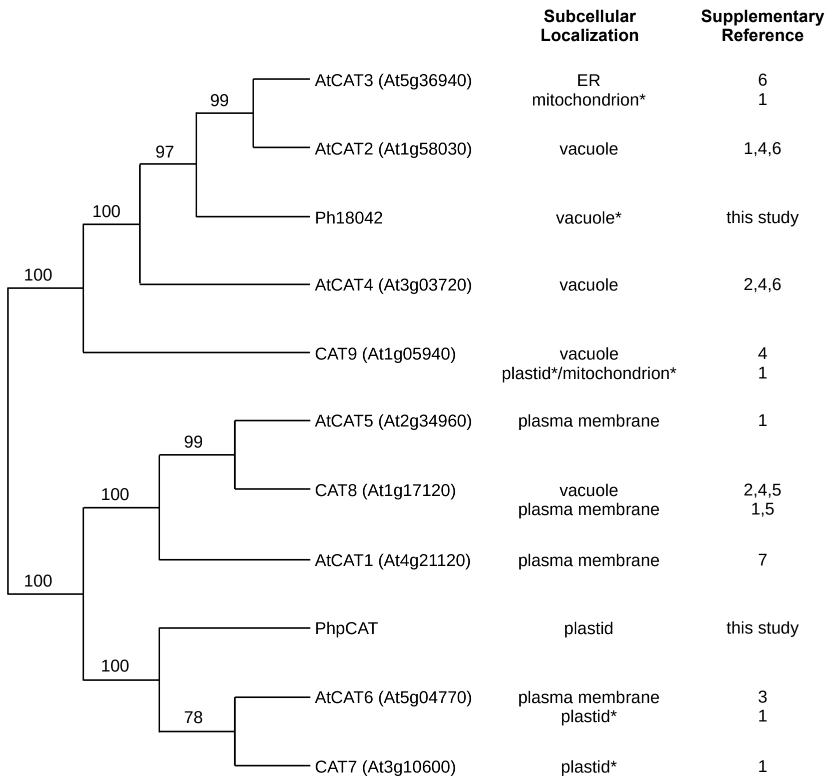

Supplementary Figure 7 | Maximum likelihood phylogenetic reconstruction of the two expressed petunia flower cationic amino acid transporters (CATs) with the Arabidopsis CATs and their subcellular localizations.

The tree was reconstructed using ClustalW and PhyML in the “A la Carte” mode of tools from the MABL phylogenetic suite (http://www.phylogeny.fr/version2_cgi/index.cgi). Numbers on branches indicate bootstrap support. Subcellular localization data comes from cited sources. Predicted localizations are indicated with an asterisk (*). At, *Arabidopsis thaliana*; CAT, cationic amino acid transporter; Ph, *Petunia hybrida*.

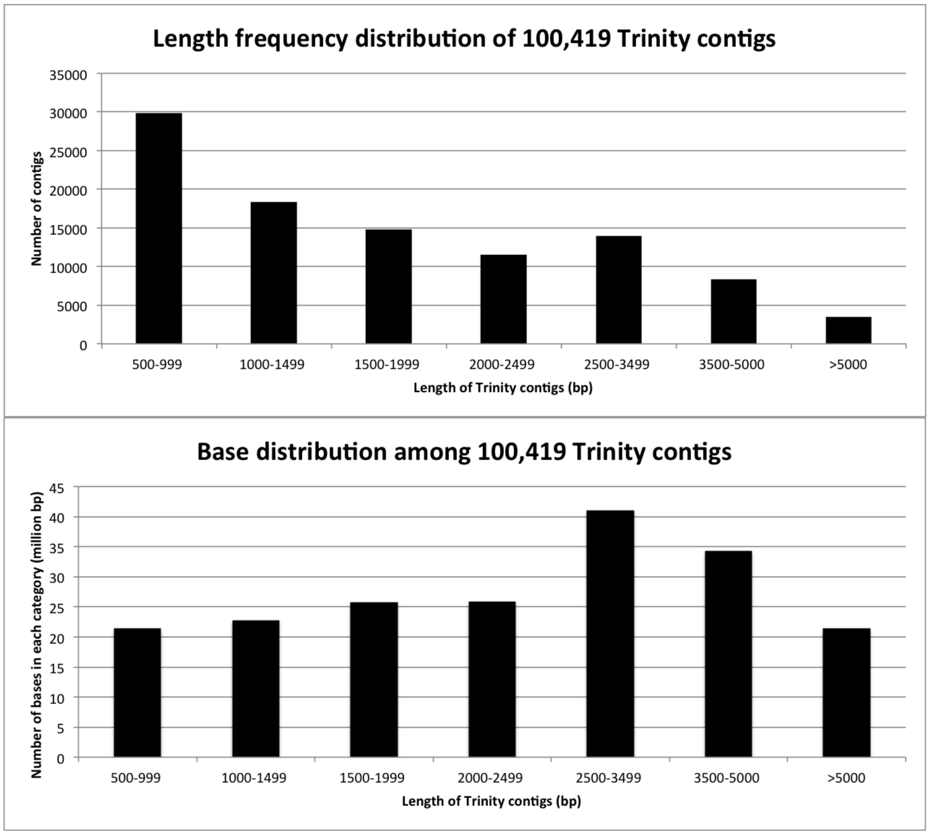

Supplementary Figure 8 | Length frequency and base distributions of Trinity contigs.

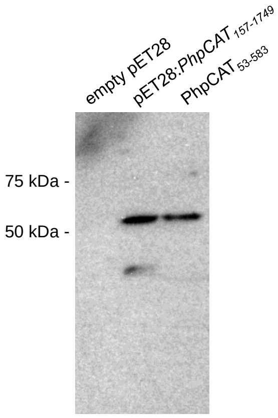

Supplementary Figure 9 | Original immunoblot image for Figure 4a.

Supplementary Table 1 | Expression of petunia *E. coli pheP* homologs in buds and day 2 postanthesis flowers.

Measurement of gene expression is based on edgeR analysis of RNA-Seq data of petunia flowers at the bud stage and on day 2 postanthesis (see Methods). Independent biological replicates (*n* = 3) are indicated as “rep 1”, “rep 2”, and “rep 3”. Petunia genes encoding homologs of *E. coli* PheP are shaded in gray. Petunia genes encoding plastidial enzymes known to be involved in phenylalanine biosynthesis, or cytosolic phenylalanine-utilizing enzymes, are shaded in green and orange, respectively. Data presented are raw read counts with CPM (counts per million reads) given in brackets, average FC (fold change) of raw read counts with edgeR FC given in brackets, and FDR (false discovery rate) values from edgeR analysis.

Contig ID	Annotation	d -1 postanthesis (bud) raw read counts (CPM)			d 2 postanthesis raw read counts (CPM)			Average FC d2 vs. d -1 (edgeR FC)	FDR value
		rep 1	rep 2	rep 3	rep 1	rep 2	rep 3		
21511	high affinity cationic amino acid transporter 1	915 (8.75)	845 (8.46)	1065 (8.76)	2089 (23.5)	2363 (24.2)	1231 (14.7)	+2.0 (+2.4)	4.26E-09
18042	cationic amino acid transporter 2	7203 (68.9)	5989 (60.0)	7192 (59.2)	14,750 (166)	13,455 (138)	11,729 (138)	+2.0 (+2.4)	6.19E-09
19221	amino acid transporter, putative	1653 (15.8)	1420 (14.2)	1562 (12.9)	813 (9.15)	861 (8.83)	856 (10.2)	-1.8 (-1.5)	8.38E-03
17220	arogenate dehydratase 1	8890 (85.0)	9742 (97.6)	12,177 (100)	131,067 (1475)	112,980 (1158)	100,609 (1198)	+11.2 (+13.5)	9.78E-63
20149	prephenate aminotransferase	6545 (62.6)	6677 (66.9)	6920 (56.9)	28,138 (317)	28,235 (289)	23,780 (283)	+4.0 (+4.8)	4.38E-26
13047	chorismate mutase 1	3700 (35.4)	4510 (45.2)	4676 (38.5)	31,459 (354)	28,783 (295)	23,440 (279)	+6.5 (+7.8)	3.00E-177
20024	phenylacetalde-hyde synthase	14,466 (138)	11,898 (119)	21,566 (177)	88,053 (991)	81,892 (840)	60,020 (715)	+4.8 (+5.8)	1.94E-32
10066	phenylalanine ammonialyase 1	24,305 (233)	24,757 (248)	28,296 (233)	140,606 (1583)	140,756 (1443)	117,317 (1397)	+5.2 (+6.2)	2.46E-34

Supplementary Table 2 | Plastidial arogenate (v_1) and cytosolic phenylpyruvate (v_2) pathway fluxes, and relative changes in flux, in flowers from control and *PhpCAT*-RNAi petunia lines at t_{0h} and t_{6h}.

	**Plastidial synthesis rate v_1 (μmol gFW^{-1} h^{-1})**			**Cytosolic synthesis rate v_2 (μmol gFW^{-1} h^{-1})**		
	t_{0h}	t_{6h}	**Relative change at t_{6h} versus t_{0h}**	t_{0h}	t_{6h}	**Relative change at t_{6h} versus t_{0h}**
**Control**	1.051 ± 0.227	0.896 ± 0.191	-14.7%	0.0067 ± 0.0017	0.161 ± 0.048	+2303%
***PhpCAT* RNAi**	0.711 ± 0.089	0.500 ± 0.065	-29.7%	0.0068 ± 0.0010	0.218 ± 0.041	+3106%
**Relative change in *PhpCAT* RNAi versus control**	-32.4%	-44.2%		1.5%	+35.4%	

(-) indicates a decrease in flux at t_{6h} versus t_{0h} or in *PhpCAT* RNAi versus control

(+) indicates an increase in flux at t_{6h} versus t_{0h} or in *PhpCAT* RNAi versus control

Supplementary References

1. Su, Y., Frommer, W. B. & Ludewig, U. Molecular and Functional Characterization of a Family of Amino Acid Transporters from Arabidopsis 1. *Plant Physiol.* **136,** 3104–3113 (2004).

2. Carter, C. *et al.* The vegetative vacuole proteome of Arabidopsis thaliana reveals predicted and unexpected proteins. *Plant Cell* **16,** 3285–3303 (2004).

3. Hammes, U. Z., Nielsen, E., Honaas, L. a., Taylor, C. G. & Schachtman, D. P. AtCAT6, a sink-tissue-localized transporter for essential amino acids in Arabidopsis. *Plant J.* **48,** 414–426 (2006).

4. Jaquinod, M. *et al.* A Proteomics Approach Highlights a Myriad of Transporters in the Arabidopsis thaliana Vacuolar Membrane. *Plant Signal. Behav.* **2,** 413–415 (2007).

5. Yang, H., Bogner, M., Stierhof, Y. D. & Ludewig, U. H+-independent glutamine transport in plant root tips. *PLoS One* **5,** (2010).

6. Yang, H., Krebs, M., Stierhof, Y.-D. & Ludewig, U. Characterization of the putative amino acid transporter genes AtCAT2, 3 & 4: The tonoplast localized AtCAT2 regulates soluble leaf amino acids. *J. Plant Physiol.* **171,** 594–601 (2014).

7. Yang, H., Postel, S., Kemmerling, B. & Ludewig, U. Altered growth and improved resistance of Arabidopsis against Pseudomonas syringae by overexpression of the basic amino acid transporter AtCAT1. *Plant. Cell Environ.* **37,** 1404–1414 (2014).
